# Inferior long-term graft survival after end-to-side reconstruction for two renal arteries in living donor renal transplantation

**DOI:** 10.1371/journal.pone.0199629

**Published:** 2018-07-11

**Authors:** Shigeyoshi Yamanaga, Angel Rosario, Danny Fernandez, Takaaki Kobayashi, Mehdi Tavakol, Peter G. Stock, Sang-Mo Kang

**Affiliations:** 1 Division of Transplant Surgery, Department of Surgery, University of California San Francisco, San Francisco, CA, United States of America; 2 Department of Surgery, Japanese Red Cross Kumamoto Hospital, Kumamoto, Japan; 3 Department of Renal Transplant Surgery, Aichi Medical University School of Medicine, Nagakute, Japan; The University of Manchester, UNITED KINGDOM

## Abstract

Living donor kidneys with two arteries can be revascularized using various techniques depending on anatomy. We hypothesized that the revascularization technique could impact long-term outcomes. We retrospectively analyzed 1714 living donor renal transplants at our institution between 1999 and 2015. Three hundred and eleven kidneys had dual arteries, and these were categorized into 5 groups; end-to-side (n = 18), inferior epigastric artery (n = 21), direct anastomosis (n = 65), side-to-side (n = 126) and ligated (n = 81). We then compared the outcomes with that of a control group (single artery, n = 1403) using Kaplan-Meier and Cox regression analyses. Cox regression was adjusted by age, sex and race/ethnicity of donor and recipient, side of kidney, transplant period and recipient surgeon. Compared to the control group, the end-to-side group had increased all-cause graft loss (10 years: 77.2% vs 24.5%, adjusted hazard ratio [aHR] 3.02, 95% confidence interval [CI] 1.30–7.03, p = 0.010) and death-censored graft loss (10 years: 82.0% vs 55.9%, aHR 4.17, 95% CI 1.63–10.68, p = 0.003), whereas the other groups did not. Our study shows that 10-year overall survival and death-censored graft survival were significantly worse for end-to-side arterial reconstruction than for other techniques. Alternative techniques to the end-to-side method should be used for accessory arteries that require revascularization.

## Introduction

In living donor renal transplantation, kidneys with multiple arteries are encountered in about 7–28% of cases [1]. Although kidneys with multiple renal arteries recovered by laparoscopic donor nephrectomy are associated with a higher risk of complications and delayed graft function than is true for kidneys with a single renal artery, long-term graft and patient survival have been reported as generally equivalent [1].

Various techniques are used to revascularize multiple arteries depending on the anatomy and institutional or surgeon preference [2, 3]. In our center, when there are two arteries and one is significantly smaller, the smaller artery is placed on the side of the larger artery, anastomosed to the inferior epigastric artery if available/suitable, or anastomosed directly to the recipient iliac arteries. For two arteries that are relatively more comparable in size, they are sewn together in a side-to-side configuration before a single anastomosis to the recipient artery is performed; alternatively, for reasons of preference or geometry, the arteries are anastomosed individually to two separate arteriotomies in the recipient artery [4].

However, whether the type of arterial reconstruction method for multiple renal arteries has an influence on long-term graft and patient survival is not known [1]. We hypothesized that the revascularization technique could have an impact on long-term graft outcome after living donor kidney transplantation. To test this hypothesis, we conducted a retrospective study of a large series of living donor transplants at our institution.

## Materials and methods

### Patient population

We retrospectively analyzed 1750 living donor renal transplantations in which kidneys were retrieved by laparoscopic donor nephrectomy at our institution between 1999 and 2015. To analyze the pure impact of each reconstruction technique, we excluded 36 cases from the analysis: 30 with more than three arteries, five with an arterial graft, and one for which recipient data was unavailable. Two artery cases (n = 311) were divided into five groups for analysis: end-to-side (n = 18), inferior epigastric artery (n = 21), direct anastomosis (n = 65), side-to-side (n = 126) and ligated (n = 81), and compared with control group (single artery, n = 1403). Demographic data were collected from patients’ electronic medical records. This study was approved by the Institutional Review Board of the University of California, San Francisco (study approval number 17–22108). The review board waived the requirement for informed consent for this research. None of the transplant donors were from a vulnerable population and all donors or next of kin provided written informed consent that was freely given.

### Details of revascularization techniques

**Fig 1:**
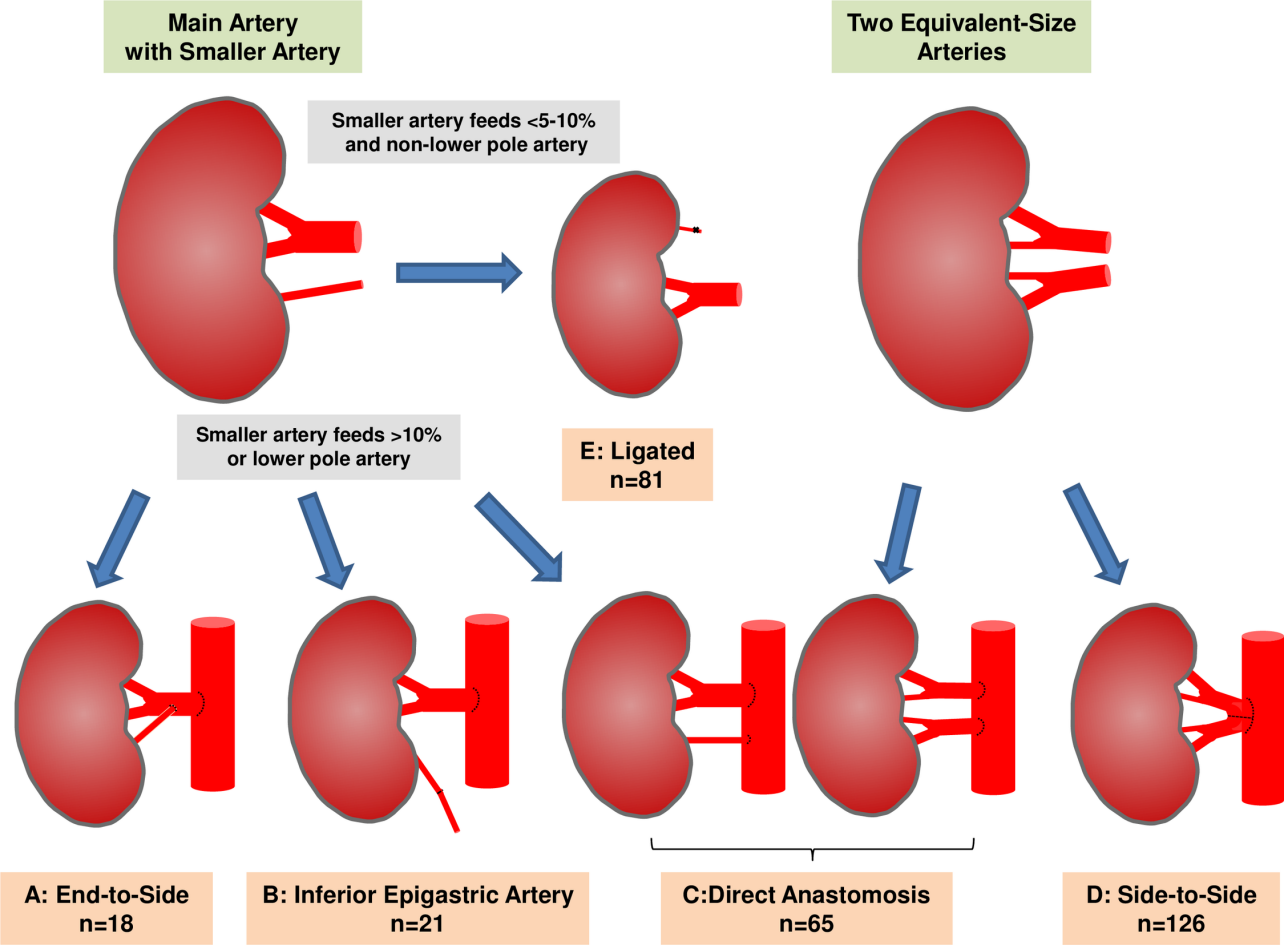
Details of revascularization techniques for the kidneys with two renal arteries.

All nephrectomies were done using an intraperitoneal pure laparoscopic donor nephrectomy approach as previously described [5–7]. The details of revascularization techniques for two arteries are shown in Fig 1. When one of the two arteries was small but could not be sacrificed (smaller artery feeds >10% or lower pole artery), revascularization was done using the (A) end-to-side reconstruction method: suturing the end of the smaller artery to the side of the dominant artery or (B) inferior epigastric artery method: smaller artery was anastomosed to the recipient inferior epigastric artery, or (C) direct anastomosis method: two arteries were anastomosed directly to the external/common iliac artery. For two relatively equivalent-size arteries, revascularization was done using the (C) direct anastomosis method as described above, or (D) side-to-side reconstruction method: two arteries are conjoined into single large orifice, allowing a single anastomosis. All ligated smaller arteries in the ligated group (E) were assumed to be feeding less than 5–10% of whole renal parenchyma; another assumption was that ligation did not affect ureteral blood supply (no lower pole artery was ligated). Estimations of the territory supplied by the smaller artery were based on pre-operative enhanced computed tomography and/or back table gross appearance of the non-washed out surface lesion after the main artery was flushed, as well as the size of the artery. Any artery less than 2 mm were generally ligated. All renal arteries were eventually anastomosed to the common/external iliac artery or aorta in an end-to-side fashion, except for the smaller renal arteries revascularized with the inferior epigastric artery. Aspirin 81 mg was routinely used perioperatively in our patients unless contraindicated.

### Statistical analysis

The Chi-squared or Fisher’s exact test was used for categorical data analysis and the Mann-Whitney U test or Kruskal-Wallis H test was used for continuous data. Post-hoc analyses for categorical and continuous data were all adjusted using the Bonferroni correction and Z-test. Two-tailed p-values ≤0.05 were considered statistically significant. Values are expressed as median [interquartile range] unless otherwise specified.

The Kaplan-Meier method was used to analyze overall graft survival (death non-censored graft survival), death-censored graft survival, and patient survival. Cox regression analysis was used to analyze the impact of each reconstruction technique on survival, adjusted for age, sex and race/ethnicity of donor and recipient, side of kidney, transplant period and recipient surgeon. Interaction analysis was done thereafter, using recipient sex and body mass index (BMI).

All analyses were done using SPSS version 22 (SPSS, Chicago, Illinois, USA). The authors have followed the suggestions of the Strengthening the Reporting of Observational Studies in Epidemiology (STROBE) statement guidelines for reporting observational studies.[8]

## Results

### Donor and recipient baseline characteristics

Baseline characteristics, including age, race, sex and BMI for both donor and recipient did not differ among donors and recipients across all the groups (Table 1). In addition, other variables, including pre-transplant dialysis, duration of dialysis therapy, original disease, ABO blood type incompatibility and re-transplant did not differ across all the groups. Length of follow-up did differ significantly and was lowest among the side-to-side group, showing that significantly higher numbers of this anastomosis type were done in the 2011–2015 period (Table 1). The lower-pole smaller arteries were identified in 66.7% (n = 12) of end-to-side cases, 90.5% (n = 19) of inferior epigastric artery cases, and 0% of ligated cases.

**Table 1:** Donor and recipient baseline characteristics.

	Single artery	Two arteries					p-value
	n = 1403	End-to-side n = 18	Inferior epigastric artery n = 21	Direct anastomosis n = 65	Side-to-side n = 126	Ligated n = 81	
Length of follow-up, months, median [IQR]	67 [33–108]	63 [23–93]	62 [25–83]	86 [40–133]	46 [24–96]*	71 [37–113]	0.004
Transplant period							0.002
1999–2005	545 (36.7)	7 (38.9)	5 (23.8)	28 (43.1)	24 (19.0)**	34 (42.0)	
2006–2010	469 (33.4)	6 (33.3)	7 (33.3)	22 (33.8)	43 (34.1)	29 (35.8)	
2011–2015	419 (29.9)	5 (27.8)	9 (42.9)	15 (23.1)	59 (46.8)**	18 (22.2)	
**Donor**							
Age, years, median [IQR]	41 [32–51]	44 [33–60]	36 [30–45]	38 [32–48]	43 [33–52]	39 [32–48]	0.264
Sex, n (%)							0.253
Male	511 (36.4)	6 (33.3)	9 (42.9)	32 (49.2)	53 (42.1)	27 (33.3)	
Female	892 (63.6)	12 (66.7)	12 (57.1)	33 (50.8)	73 (57.9)	54 (66.7)	
BMI, kg/m^2^, median [IQR]	26.0 [23.4–29.4]	25.7 [23.4–28.9]	25.1 [24.3–28.2]	27.0 [24.2–29.6]	26.4 [24.0–29.3]	25.6 [22.7–27.4]	0.405
Afro-American race, n (%)	79 (5.6)	1 (5.6)	1 (5.6)	3 (4.6)	11 (8.7)	4 (4.9)	0.794
Side of kidneys, n (%)							0.118
Left	1115 (79.5)	17 (94.4)	20 (95.2)	49 (75.4)	94 (74.6)	61 (75.3)	
Right	288 (20.5)	1 (5.6)	1 (4.8)	16 (24.6)	32 (25.4)	20 (24.7)	
Donor S-Cr at baseline,mg/dl, median [IQR]	0.78 [0.66–0.90]	0.76 [0.67–0.90]	0.78 [0.63–0.99]	0.81 [0.70–1.00]	0.76 [0.67–0.90]	0.72 [0.63–0.83]	0.119
**Recipient**							
Age, years, median [IQR]	46 [33–56]	51 [36–62]	45 [38–62]	40 [31–54]	49 [33–58]	45 [36–54]	0.385
Sex, n (%)							0.941
Male	821 (58.5)	10 (55.6)	11 (52.4)	37 (56.9)	69 (54.8)	45 (55.6)	
Female	582 (41.5)	8 (44.4)	10 (47.6)	28 (43.1)	57 (45.2)	36 (44.4)	
BMI, kg/m^2^, median [IQR]	25.8 [22.3–29.9]	25.8 [22.8–32.5]	22.7 [21.2–30.6]	24.8 [22.2–28.7]	24.9 [22.3–30.1]	25.8 [23.0–29.2]	0.365
Pre-transplant dialysis, n (%)	969 (69.1)	12 (66.7)	12 (57.1)	46 (70.8)	81 (64.3)	49 (60.5)	0.416
Dialysis duration, months, median,[IQR]	16 [9–30]	18 [8–31]	17 [9–38]	15 [6–28]	14 [9–28]	13 [8–28]	0.712
Afro-American race, n (%)	103 (7.3)	1 (5.6)	2 (9.5)	7 (10.8)	11 (8.7)	4 (4.9)	0.803
Original disease							
DM, n (%)	295 (21.0)	3 (16.7)	7 (33.3)	13 (20.0)	28 (22.2)	14 (17.3)	0.707
FSGS, n (%)	83 (5.9)	1 (5.6)	1 (4.8)	5 (7.7)	12 (9.5)	5 (6.2)	0.715
ABO blood type incompatible, n (%)	15 (1.1)	0 (0)	0 (0)	0 (0)	3 (2.4)	0 (0)	0.533
Re-transplant, n (%)	126 (9.0)	2 (11.1)	4 (19.0)	3 (4.6)	10 (7.9)	7 (8.6)	0.490
Pediatric transplant. n (%)	90 (6.4)	0 (0)	0 (0)	5 (7.7)	10 (7.9)	5 (6.2)	0.647

* p = 0.046 adjusted post-hoc analysis, (vs single artery).

** Z-test p<0.05 adjusted by Bonferroni correction (vs single artery).

Abbreviations: BMI, body mass index; DM, diabetes mellitus; FSGS, focal segmental glomerulosclerosis; IQR, interquartile range; S-Cr, serum creatinine.

### Recipient outcomes after kidney transplantation

The groups did not significantly differ with respect to the frequency of acute rejection and delayed graft function. The end-to-side group had the highest rate of ureteral complications (n = 3, 16.7%). Critical graft thrombosis causing immediate graft loss (complete arterial thrombosis and vein thrombosis) occurred only in the direct anastomosis and side-to-side groups, as well as the control group (Table 2). Partial arterial thrombosis occurred 5.6% in the end-to-side group, 0% in the inferior epigastric artery group and 0.1% in the control group.

**Table 2:** Recipient outcomes after kidney transplantation.

	Single artery	Two arteries					p-value
	n = 1403	End-to-side n = 18	Inferior epigastric artery n = 21	Direct anastomosis n = 65	Side-to-side n = 126	Ligated n = 81	
Cumulative acute rejection, n (%)	271 (19.3)	4 (22.2)	4 (19.0)	13 (20.0)	23 (18.3)	12 (14.8)	0.944
Artery thrombosis, n (%)							<0.001
Whole	2 (0.1)	0 (0)	0 (0)	1 (1.5)	0 (0)	0 (0)	
Partial	1 (0.1)	1 (5.6)*	0 (0)	1 (1.5)*	0 (0)	0 (0)	
Vein thrombosis, n (%)	3 (0.2)	0 (0)	0 (0)	1 (1.5)	2 (1.6)	0 (0)	
Delayed graft function, n (%)	41 (2.9)	1 (5.6)	0 (0)	4 (6.2)	5 (4.0)	2 (2.5)	0.604
Ureteral complication, n (%)	67 (4.8)	3 (16.7)	1 (4.8)	5 (7.7)	7 (5.6)	4 (4.9)	0.281

*Z-test p<0.05 after Bonferroni correction (vs single artery).

### Risk analyses for each arterial reconstruction technique

**Fig 2:**
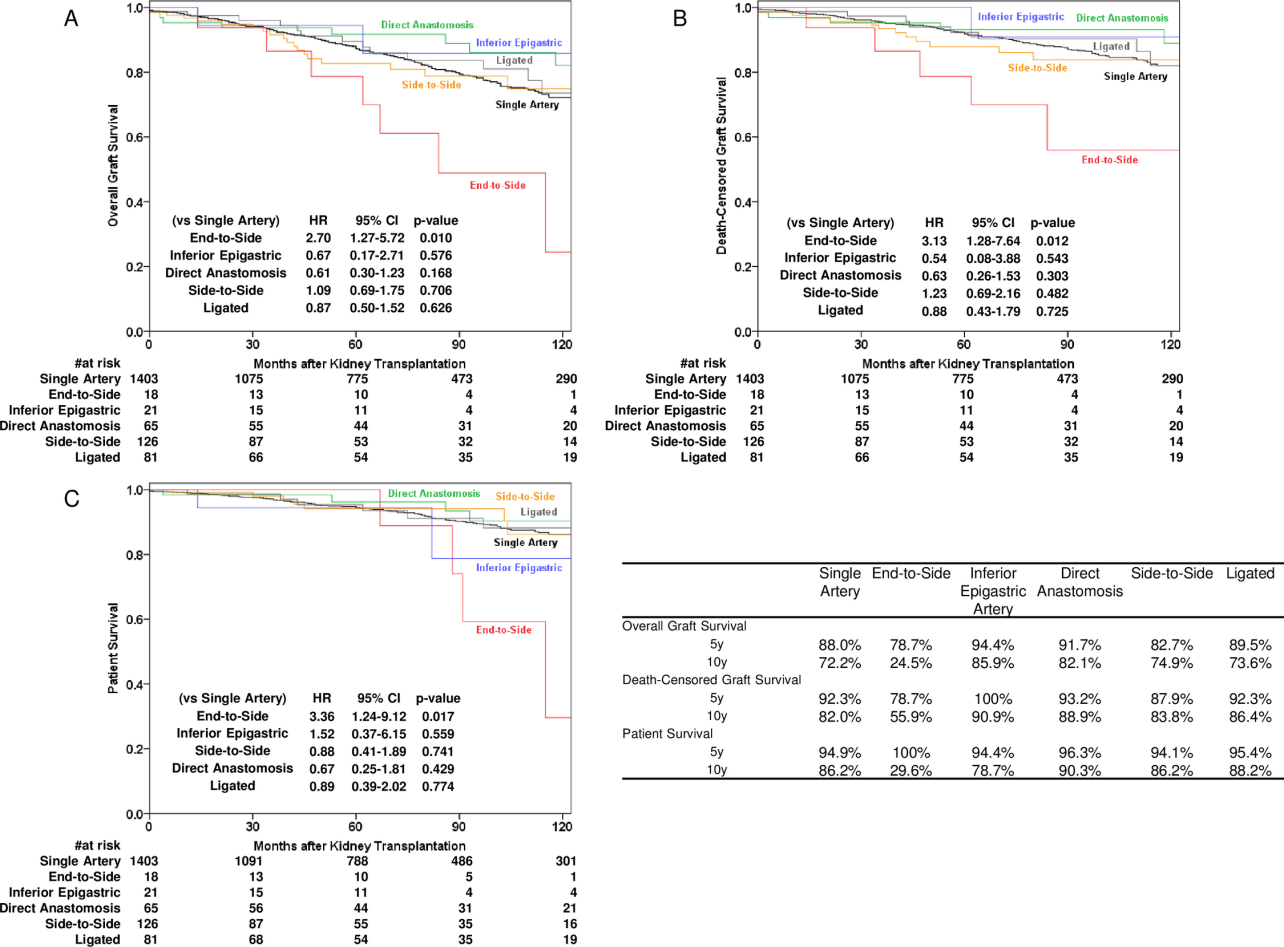
**Kaplan-Meier curve for (A) overall graft survival, (B) death-censored graft survival and (C) patient survival after living donor kidney transplantation.** Abbreviations; HR: hazard ratio, CI: confidence interval.

The 10-year survival rates for the end-to-side group were significantly lower than those for the control group for overall graft survival (24.5% vs 72.2%, p = 0.007), death-censored graft survival (55.9% vs 82.0%, p = 0.008) and patient survival (29.6% vs 86.2%, p = 0.012); this was not true for the other techniques (Fig 2). The alternative reconstruction techniques for smaller arteries—inferior epigastric artery revascularization and direct anastomosis—showed equivalent 10-year overall graft survivals to control kidneys (inferior epigastric artery revascularization: 85.9% and direct anastomosis: 82.1%).

**Fig 3:**
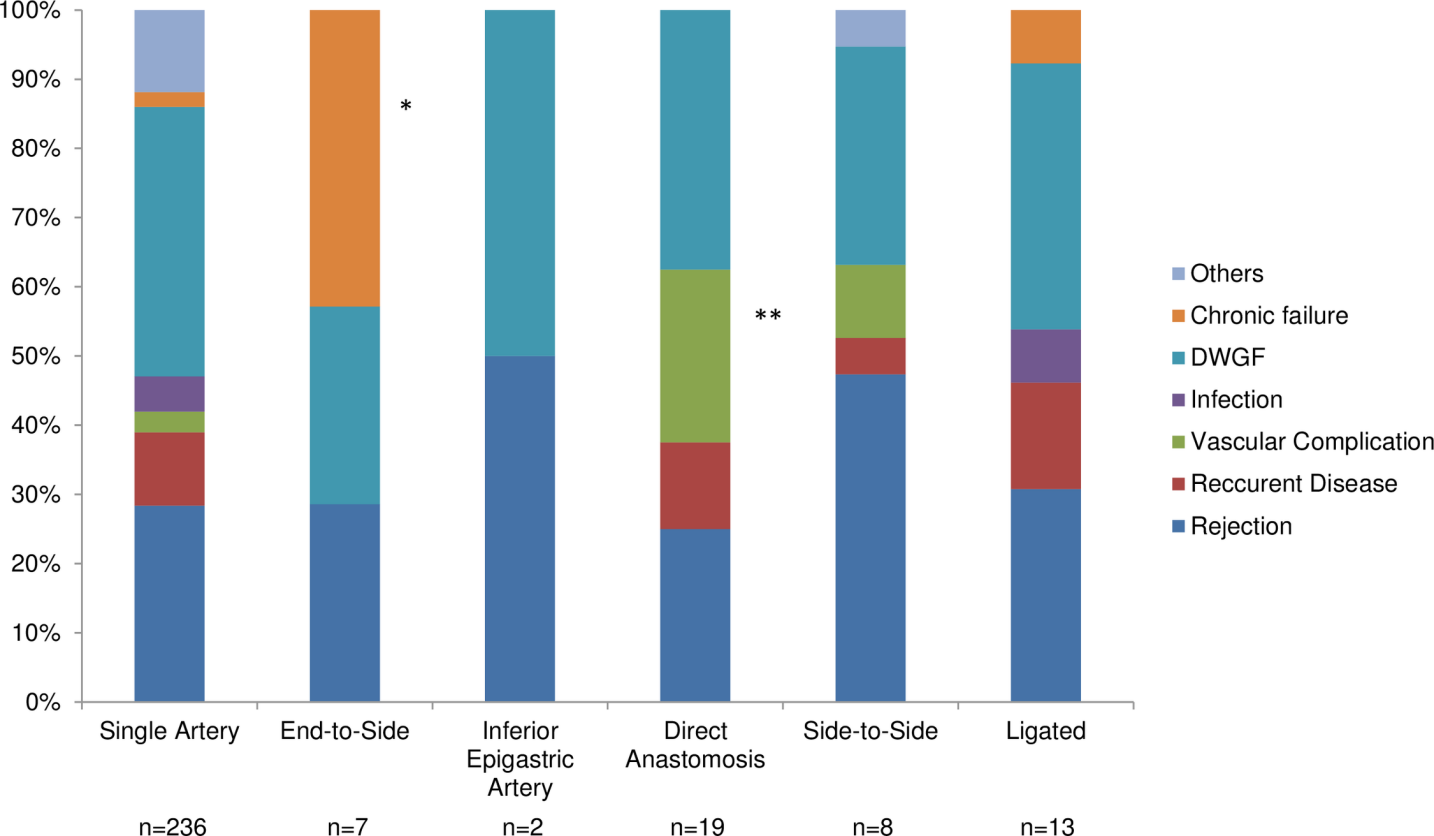
Reasons for graft loss within 10 years after living donor kidney transplantation. *Z-test p<0.05 after Bonferroni correction for chronic failure (vs single artery). ** Z-test p<0.05 after Bonferroni correction for vascular complication (vs single artery). Abbreviation: DWGF, death with graft functioning.

**Fig 4:**
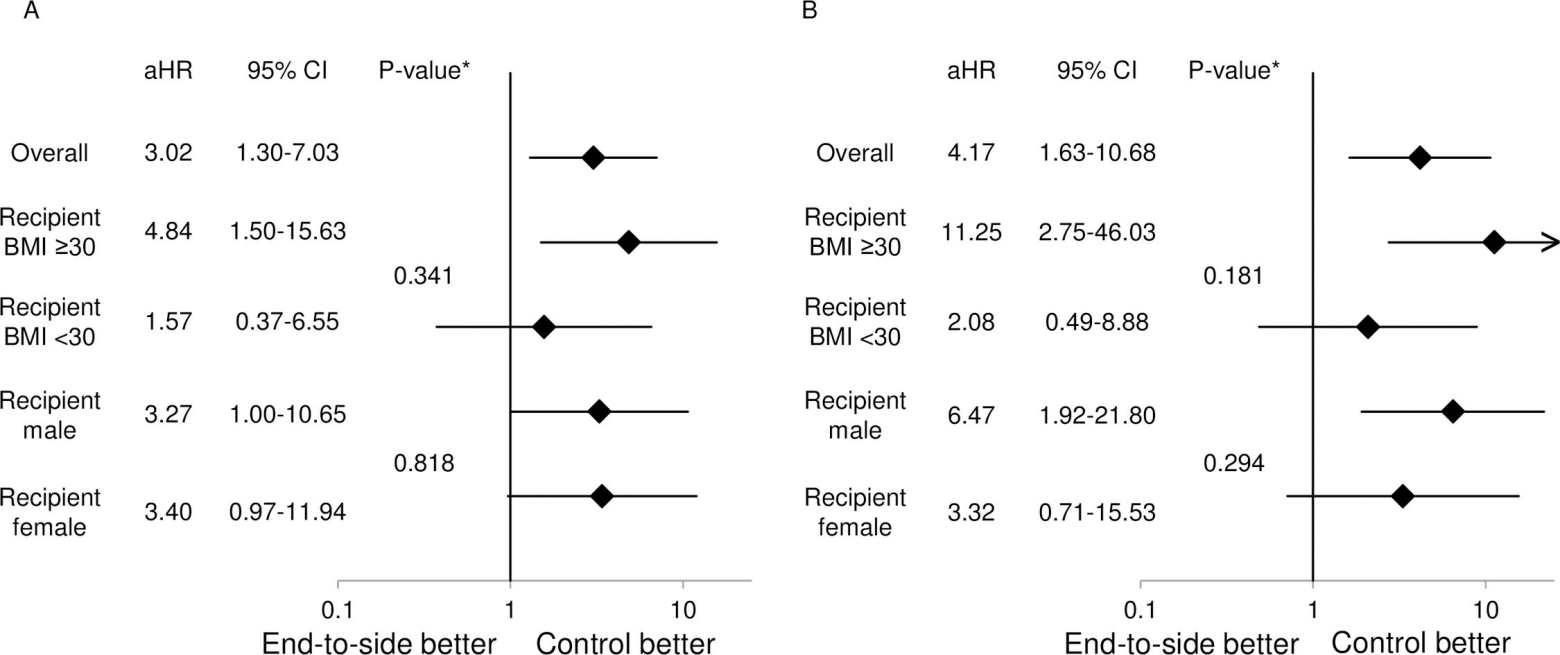
**Interaction analyses for the risks of end-to-side anastomosis for (A) all-cause graft loss and (B) death-censored graft loss.** *P-value for interaction. Abbreviations: aHR, adjusted hazard ratio; BMI, body mass index; CI, confidence interval.

The causes of graft loss within 10 years are shown in Fig 3. The end-to-side group had a significantly higher incidence of non-specific chronic failure (including fibrosis and de novo focal segmental glomerulosclerosis) than control. After multivariate adjustment for age, sex and race/ethnicity of donor and recipient, side of kidney, transplant period and recipient surgeon, the end-to-side group had a significantly higher adjusted hazard ratio for all-cause graft loss, as well as death-censored graft loss, when compared to control (Table 3). Furthermore, interaction analyses showed that the impact of end-to-side anastomosis on all-cause graft loss and death-censored graft loss was greater in recipients who had a BMI ≥30 and in male recipients (Fig 4).

**Table 3:** Adjusted risk analysis of all-cause graft loss, death-censored graft loss and death.

(vs Single artery)	All-cause graft loss			Death-censored graft loss			Death		
	aHR	95% CI	p-value	aHR	95% CI	p-value	aHR	95% CI	p-value
End-to-side	3.02	1.30–7.03	0.010	4.17	1.63–10.68	0.003	2.04	0.62–6.72	0.243
Inferior epigastric artery	0.75	0.19–3.06	0.691	0.79	0.11–5.74	0.818	1.38	0.33–5.81	0.664
Direct anastomosis	0.64	0.31–1.32	0.230	0.56	0.23–1.38	0.206	1.03	0.37–2.88	0.952
Side-to-side	0.97	0.58–1.62	0.911	1.13	0.61–2.11	0.697	0.76	0.33–1.75	0.516
Ligated	0.79	0.44–1.41	0.417	0.77	0.36–1.64	0.491	0.99	0.43–2.29	0.986

Adjusted with recipient/donor age, sex and Afro-American race, side of kidney, transplant period and recipient surgeon.

Abbreviations: aHR, adjusted hazard ratio; CI, confidence interval.

## Discussion

To our knowledge, this is the first report showing significantly inferior long-term graft survival of a specific arterial reconstruction method—end-to-side—for living donor kidneys with two arteries. Long-term graft survival was previously reported to be similar for single and multiple arteries [2, 4, 9–20]; however, most studies did not analyze the impact of each reconstruction technique and included deceased donor kidneys with an aortic patch. Only one previous report has addressed the graft survival of end-to-side reconstruction of renal artery [15]. Although that study lacked the power to find statistically significant differences, the end-to-side group showed a markedly lower 5-year graft survival of about 40% (vs 70% for side-to-side group and 90% for direct anastomosis group), which would support the hypothesis of our study.

In our study, arterial thrombosis rates were lower than reported by other groups [1]. Our center has been performing approximately 300–350 kidney transplants per year, and is one of the highest volume centers in the US. Furthermore, we use aspirin routinely in our renal transplant patients, which may also contribute to the low rate of thrombosis. Although the potential reasons for the low thrombosis rates in our center are well beyond the scope of this study, the low thrombosis rates would be predicted to add validity to this study, by minimizing this potentially confounding variable.

A unique aspect of the end-to-side technique is that the main artery diameter is presumably sufficient to supply the original distribution; the main artery of the kidney with two arteries is smaller than that of the kidney with a single artery. Adding another smaller artery upstream of this main artery is technically difficult and would potentially cause stenosis and thrombosis in the smaller artery. The end-to-side technique is also more vulnerable to stenosis, as the diameter of the larger artery is generally smaller and needs to supply the entire kidney. This contrasts with the inferior epigastric reconstruction or separate anastomosis techniques, which provide an independent blood supply, and the larger artery only needs to supply its original portion of the kidney. Furthermore, the thrombus and/or hyperplasia of the smaller artery could spread in the main artery, producing future stenosis and chronic ischemia of the main artery as well. However, without angiography, the relative contribution of thrombosis or stenosis cannot be determined. Importantly, post-transplant renal artery stenosis is strongly associated with inferior overall graft survival [21].

Several ancillary findings in our study support the concept of arterial insufficiency and/or insufficient renal mass as a contributor to the increased graft loss in end-to-side reconstruction. First, a significantly higher incidence of non-specific chronic failure was observed among the reasons for graft loss for end-to-side group, possibly due to the chronic ischemic injury to the graft. Secondly, adjusted hazard ratios of all-cause graft loss and death-censored graft loss were higher in patients who would benefit from more renal mass, namely male patients and patients with BMI ≥30.

Both the inferior epigastric artery method and the direct anastomosis to recipient iliac artery appear to be good alternatives to the end-to-side method because long-term graft survival was similar to that in the control group. The inferior epigastric artery is often available and can be anastomosed after the main renal artery is reperfused, which contributes to reduced ischemia time and reduced incidence of delayed graft function [22]. However, the inferior epigastric artery is not always available because of atherosclerotic disease or insufficient size relative to the accessory renal artery [23]. Further prospective studies would be needed to compare the two methods directly but would be difficult to conduct.

A recent study of 27 kidneys [24] with ligation of a tiny accessory artery (feeds <5% of parenchyma and non-lower pole artery) reported favorable short-term outcomes, including estimated glomerular filtration rate four years after living donor kidney transplantation. The mean diameter of the ligated artery was 1.82 mm in that study. The authors concluded that less than 5% of infarction had no impact on graft function and that arteries < 2 mm could be safely ligated to avoid arterial complications. Our study adds to those findings in showing that the 10-year graft and patient survival of kidneys with a ligated artery were similar to those with a single artery.

Our study has the usual limitations of a retrospective, single-institution series. Additional limitations included the lack of immunosuppressive drug information (type, adherence and treatment for rejection) for recipients. The cumulative incidence of acute rejection was the same among the groups, but we could not exclude the possible confounding effect due to long-term immunological damage. However, the cause of graft loss was not clearly immunologic as discussed above. In general, most kidney transplant patients at our center receive aspirin perioperatively, especially after any complex vascular reconstruction, unless there is a contraindication. We do not use heparin or any other form of anticoagulation on a routine basis. Further statistical analysis such as propensity-score matching to mitigate the long-term survival data was not done because the baseline characteristics of the five groups were all similar to those of the control group.

In conclusion, our study shows that 10-year overall survival and death-censored graft survival were significantly worse for end-to-side arterial reconstruction than for single artery group. While we acknowledge the pitfalls of retrospective surgical studies, these types of data are not available in registry data. Our results clearly suggest that, if possible, the end-to-side reconstruction technique should not be used. Further prospective and multi-center studies would be ideal but are extremely difficult to conduct; however, addition of more granular reconstruction data in large databases such as the SRTR database may be useful to examine whether outcomes differ for specific arterial reconstruction methods for kidneys with two arteries.

## Abbreviations

aHR: adjusted hazard ratio
BMI: body mass index
CI: confidence interval
